# Remarks Tending to Elucidate the History of Erysipelas, and Especially of Phlegmonous Erysipelas

**Published:** 1831-08

**Authors:** 

**Affiliations:** Physician to La Charité.


### ERYSIPELAS.

*Remarks tending to elucidate the History of Erysipelas, and especially of Phlegmonous Erysipelas.* By Dr. Corbin, Physician to La Charité. (*Journ. Complement.* abridged.)

The principal objects Dr. Corbin has in view, are to offer a few comments upon certain forms of erysipelas which appear to deserve especial attention; to describe the state of the gastro-intestinal mucous membrane, in a certain number of persons who died of the disease; and also to adduce some examples in which erysipelas exerted a remarkable influence over other concomitant maladies.

Phlegmonous erysipelas is frequently complicated with gangrene, but the latter condition arises in different ways. Mortification of the skin is generally consecutive to destruction of the subcutaneous cellular tissue: and in this case we can detect, before gangrene takes place, some signs of fluctuation; and, if the disease be abandoned to its course, ulceration occurs, and shreds of the cellular tissue, mingled with pus, are discharged; the external integuments are destroyed, and the muscles or aponeuroses are laid bare. In such instances, free incisions, if made at an early period of the disease, may prevent mortification, and, if at a later stage, they may limit its extension. The parts do not assume a black appearance, nor is there any gangrenous odour. In other, and less common cases, to which alone ought to be applied the term of gangrenous erysipelas, the gangrene commences in the skin, and is preceded by the appearance of phlyctænæ, or by the black and livid tint, and peculiar odour, of this class of disease. Here incisions have always appeared useless, and, when they have been made, the edges of the wounds suffered more from gangrene than the other parts. Erysipelas of this last-mentioned species is usually very severe: it is characterized from the commencement by prostration of strength, and is almost always quickly fatal. Such cases are, fortunately, rare. Thus, in erysipelas of the limbs, in nine cases out of ten, gangrene takes place after the suppuration and destruction of the cellular tissue. We frequently see pure phlegmonous erysipelas arise in the scrotum, and a part of it destroyed by mortification. Upon the face and hairy scalp, gangrenous erysipelas (confining the term to its proper limits,) is rarely seen; and, when it does occur, the skin always mortifies after suppuration of the subcutaneous cellular tissue. This fact is illustrated by the following case, which is also interesting in some other respects.

A man, forty-eight years of age, after having drank very hard, received a sabre wound, three inches long, upon the left parietal bone. He was admitted the next day (March 3d,) into the Hôtel Dieu. Attempts had already been made to promote the immediate reunion of the divided parts, although, from the appearance of the wound, they were not likely to succeed. The adhesive plasters, which had been employed, were removed, and the lips of the wound were separated: the bone was not exposed, but at the anterior part of the wound, which approached the coronal suture, the fibres of the aponeurosis of the occipito-frontalis muscle were denuded. The edges of the wound were laid very gently together, and, as erysipelas was at that time very common, cold lotions were ordered, and twenty leeches were applied to the neck.

On the 4th, the face was rather swollen.

5th. Slight erysipelatous appearance on the right eye. Cold lotions continued; blister to the neck. The erysipelas continued to extend, but, before it had reached its highest degree of severity, it disappeared entirely.

On the 9th, there was seen at the bottom of the wound a yellow-looking substance, which was found to be the aponeurosis already mortified. The patient complained of being dull and heavy, and of a sensation of weight in the head, which was so painful that he could scarcely bear to rest upon his pillow.

11th. Manifest fluctuation at the back part of the scalp, and extensive separation of the scalp from the bones. The abscess was opened, and a small quantity of pus was discharged. Much relief followed, and the following days no remarkable symptoms occurred. Mortification of the aponeurosis still continued, and shreds of yellowish-looking fibres were, from time to time, separated from it. The patient still continued drowsy, but could obtain no sound sleep. Inflammation of the membranes of the brain, with violent fever, convulsions, and great prostration of strength, soon came on, and the patient sank, and died in a few days.

Upon dissection, the lungs were found engorged with blood, and appearances of chronic gastro-enteritic inflammation were detected. Upon the outside and upper part of the cranium, the cellular tissue between the bones and the aponeurosis was entirely destroyed. The surface of the bones was covered with a sanious discharge, and exfoliation of them had commenced. The pia mater was inflamed and thickened, and between this membrane and the arachnoid there was an effusion of pus.

This case is remarkable, not only on account of the progress of the erysipelatous inflammation, but it shews also that a wound on the head may remain open, although the bones are not denuded. If the occipito-frontal aponeurosis is exposed, it exfoliates like a tendon, and often in a very gradual manner.

Cases of erysipelas are occasionally seen, which appear to be intermediate between the superficial and phlegmonous forms of the disease. In such instances, there is but little swelling, no collection of matter, to any great extent, under the skin, but here and there small insulated abscesses. In a patient named Mainvielle, after an attack of erysipelas of the face and scalp, several small abscesses formed in the neck, behind the ears, and upon the cranium. In another patient, after a similar attack, accompanied with enormous swelling of the face and scalp, an abscess formed upon the right upper eyelid, and numerous small collections of matter, from the size of a filbert to that of a cherry-stone, also formed upon the scalp. These abscesses remained for a long time very hard; some of them disappeared spontaneously, and others were opened, and healthy pus was discharged from them.

External erysipelas frequently disappears from one part, while at the same time the disease attacks a more or less distant region of the body. Thus, in a man named Tessier, who was admitted into the Hôtel Dieu, erysipelas of the leg and foot disappeared when the face became the seat of the disease, and the parts originally affected were again attacked when the face recovered. Erysipelas also frequently exercises a revulsive influence upon internal diseases. A young man was attacked with acute pulmonary catarrh: he was bled frequently, but without decided advantage; he was much oppressed, skin hot, pulse hard and quick. He was attacked with erysipelas of the nose, which quickly extended to the face and scalp. The feverish symptoms increased, and he became delirious. Leeches were applied to the neck, and he was bled in the foot; in a few days the erysipelas disappeared. From the time that the external inflammation appeared, and while it lasted, the patient breathed freely; there was less expectoration, and, in fact, there was every reason to believe that the bronchitic affection had ceased. It might at first have been presumed that this diminution of the symptoms depended as much upon the repeated abstraction of blood, as upon any revulsive influence of the external inflammation: but no sooner had the erysipelas ceased, than the cough, oppressed breathing, and other indications of bronchitis, reappeared with increased severity, and it was again necessary to have recourse to venesection.

Phlegmonous erysipelas of the lower extremities, of the most severe kinds, is very often produced by the slightest external causes: either from excoriations, slight wounds, the neglect of old ulcers, or by applying stimulating remedies to them; and sometimes simple contusions are sufficient to produce the disease, as in a man named Wivet, among other similar instances, who died in three days of erysipelas, in consequence of falling upon his knee. Sometimes no external cause can be detected.

When we oppose to these cases numerous instances of other patients, placed in the same circumstances, or even affected, during the same season, with much more severe external lesions, and in whom, notwithstanding, no erysipelatous disease is developed, we must presume that, in the former, there existed some peculiar disposition, or, to speak less vaguely, a lesion of some important organ, and particularly of the abdominal viscera. This idea is confirmed when we detect a red and dry tongue, headach, sensibility in the epigastrium, an inflated state of the belly, diarrhœa, or enlargement of the liver: but the results of dissection in eleven fatal cases of erysipelas, afford the most satisfactory proof of the accuracy of this opinion.

In the first, a patient named Tupin, the mucous membrane of the stomach was nearly of a black colour, and softened throughout the region of the pylorus: the commencement of the duodenum exhibited the same appearances. Almost the whole of the small intestines, to within an inch above the cœcum, was of a deep violet colour. In the large intestines, some of the glands were hypertrophied, and appeared like small pustules.

In the second case, a patient named Pflüg, stomach highly coloured in different parts, with patches of a red and brown appearance; the colon, throughout its whole extent, of a deep red colour.

Case III. Duranton: Appearances of chronic inflammation of the stomach and duodenum, characterised by reddish tubercles; rectum distended with fæces.

Case IV. Lherbier: Peritoneum of a red colour, with serum effused. Stomach, patches of brown and black colour. Duodenum, a circular ulceration, about the size of half a crown.

Case V. Scheier: Mucous membrane of the stomach softened, and of a dark slate colour; the submucous cellular tissue of the duodenum deeply injected; red patches in the cæcum.

Case VI. Wivet: The mucous membrane of the stomach and duodenum softened, and of a grey colour, throughout nearly its whole extent.

Case VII. Debry: In the stomach, near the cardiac orifice, was found a smooth hollow tumor, the size of a small renette apple, containing bloody serum. The whole surface of the stomach of a dark colour.

Case VIII. Tronnet: The mucous membrane of the stomach softened, and generally pale; small red spots on the great curvature.

Case IX. Lefebre: Liver studded with grey tubercles; spleen softened, and of a large size.

Case X. Lambert: The mucous membrane of the stomach of a deep slate colour.

Case XI. Delgutte: Biliary calculi were found.

Thus, with the exception of the last cases, in which no striking morbid appearances were detected, in all the bodies there were considerable lesions of the abdominal organs. If we compare these results with our observations during life, it will be difficult to deny that most cases of erysipelas depend upon some internal cause. If such be the most frequent cause of erysipelatous diseases, it may appear singular to attribute to these maladies a decided influence over the progress of internal inflammations. This influence is, however, very evident, and not more astonishing than other revulsions affected by nature or art. No fact is better ascertained than this kind of antagonism, which is established, in certain cases, between the skin and internal mucous membranes, and especially the gastro-intestinal. Hence the use of purgatives and emetics in the treatment of erysipelas: but these means should only be employed when the digestive powers are healthy, or at least when there is simple obstruction of the stomach and bowels. In similar instances to those above described, we must, it is true, act principally upon the abdominal organs, but antiphlogistic and emollient remedies can alone be employed with safety.

